# Shen Qi Wan Ameliorates Learning and Memory Impairment Induced by STZ in AD Rats through PI3K/AKT Pathway

**DOI:** 10.3390/brainsci12060758

**Published:** 2022-06-09

**Authors:** Junhao Huang, Zhiwei Xu, Hongshu Chen, Yiyou Lin, Jiale Wei, Sichen Wang, Hongxia Yu, Shuo Huang, Yehui Zhang, Changyu Li, Xiaojie Zhou

**Affiliations:** 1 School of Pharmaceutical Sciences, Zhejiang Chinese Medical University, Hangzhou 310053, China; hjhjyhh@163.com (J.H.); xuzhiwei@zjams.com.cn (Z.X.); linyiyou@163.com (Y.L.); yhj112026@163.com (J.W.); cherryw998@sina.com (S.W.); ytgx163@163.com (H.Y.); hs6.5@163.com (S.H.); iris950903@163.com (Y.Z.); 2 The First Affiliated Hospital of Zhejiang Chinese Medical University, Hangzhou 310006, China; chenhongshu1981@sina.com; 3 Academy of Chinese Medical Sciences, Zhejiang Chinese Medical University, Hangzhou 310053, China

**Keywords:** Alzheimer’s disease, Shen Qi Wan, PI3K/AKT pathway, learning and memory

## Abstract

Alzheimer’s disease is the most common form of neurodegenerative disease, and increasing evidence shows that insulin signaling has crucial roles in AD initiation and progression. In this study, we explored the effect and underlying mechanism of SQW, a representative formula for tonifying the kidney and promoting yang, on improving the cognitive function in a streptozotocin-induced model of AD rats. We investigated memory impairment in the AD rats by using the Morris water test. HE and Nissl staining were employed to observe the histomorphological changes in the hippocampal. Expression levels of NeuN and proteins related to Tau and apoptosis were measured using immunohistochemistry and Western blotting, respectively. Additionally, we performed RNA sequencing, and the selected hub genes were then validated by qRT-PCR. Furthermore, the protein expression levels of PI3K/AKT pathway-related proteins were detected by Western blot. We found that SQW treatment significantly alleviated learning and memory impairment, pathological damage, and apoptosis in rats, as evidenced by an increased level of NeuN and Bcl-2, and decreased phosphorylation of Tau, Bax, and Caspase-3 protein expression. SQW treatment reversed the expression of insulin resistance-related genes (Nr4a1, Lpar1, Bdnf, Atf2, and Ppp2r2b) and reduced the inhibition of the PI3K/AKT pathway. Our results demonstrate that SQW could contribute to neuroprotection against learning and memory impairment in rats induced by STZ through activation of the PI3K/AKT pathway.

## 1. Introduction

Alzheimer’s disease (AD), an age-related neurodegenerative disease, is characterized by progressive cognitive and memory impairment and is estimated to affect 131.5 million by 2050 [1]. Currently, the accumulation of amyloid-beta (Aβ) plaques and the formation of neurofibrillary tangles, neuroinflammation, tau protein hyperphosphorylation, and oxidative stress in the cerebral cortex and hippocampus were considered to be responsible for the occurrence and development of AD [1,2,3,4]. As an incurable disease, it has brought a heavy burden to society, families, and individuals [5]. Unfortunately, there are no effective therapeutic strategies for reversing AD disease progression. Therefore, a better understanding of the pathogenesis of AD and further developing potential drugs have vital clinical significance for AD patients.

More and more studies have shown that AD is closely related to type 2 diabetes mellitus (T2DM), and 80% of senile dementia patients suffer from T2DM, with varying degrees of insulin resistance (IR), so senile dementia is also known as type 3 diabetes mellitus [6,7]. IR is considered to be a disorder of the insulin signaling pathway, which significantly causes Aβ deposition, hyperphosphorylation of Tau protein, and impaired synaptic function, and then affects cognitive functions in the brain [8,9]. The phosphatidylinositol 3-kinase/protein kinase B (PI3K/AKT) signaling pathway, a classic insulin resistance-related pathway, has been shown to play an essential role in the central nervous system [10,11].

PI3Ks are involved in intracellular signal transduction and cell survival regulation, and are a key element upstream of the PI3K/AKT signaling pathway [12]. AKT plays a role in regulating cell growth and migration under the action of PI3K-dependent kinase (PDK) [13,14]. GSK3β is one of the main downstream target molecules of AKT, and it is involved in the activation of apoptosis factors, amyloid precursor protein (APP), and Tau protein [15,16,17]. The PI3K/AKT/GSK3β signaling pathway is related to the morphological and functional changes of AD neurons. It can maintain the survival of neural stem cells and affect the occurrence and development of AD. In view of the crucial roles of the insulin resistance pathway, namely the “PI3K-AKT pathway” in neurodegenerative encephalopathies such as senile dementia, further exploration of the relationship between T2DM and AD has new value in AD treatment.

In the theory of traditional Chinese medicine, Alzheimer’s disease is classified as “dementia”, “stupidity”, “forgetfulness”, and other diseases; these diseases belong to the diseases of the elderly and are closely linked to the deficiency of kidney essence and deficiency of kidney yang [18]. Kidney essence is considered to be the source of human life, and its source and function are mainly or partially reflected in the dynamic balance of stem cells, microenvironments, and the “neuro-endocrine-immune network” [19]. According to the theory of Tibetan imagery in TCM, “the kidneys govern the storage of essence, and the essence and marrow are connected to the brain”, and “the brain is the sea of marrow” [20]. If the kidney essence is deficient, the brain will become malnourished, and over time, the bone marrow fails and the brain weakens, leading to dementia, muscle stiffness, and movement disorders, with symptoms and signs similar to neurodegenerative diseases [21,22]. At present, kidney deficiency is considered to be an important pathological basis for AD pathogenesis [22].

Shen Qi Wan (SQW) is a traditional Chinese herbal formula consisting of eight herbs: Radix Rehmanniae, Chinese Yam, Cornus officinalis, Alisma Orientale, Poria Cocos, Cortex Moutan, Cassia Twig, and Aconite, and it is recorded in the “Synopsis of Prescriptions of the Golden Chamber” [23]. SQW has been used effectively for hundreds of years to treat a variety of disorders associated with kidney yang deficiency syndrome [24,25]. Modern pharmacological studies have shown that SQW has pharmacological effects such as enhancing the adrenal cortex axis, thyroid axis, and gonadal axis; delaying aging; regulating immune function; and anti-oxidation [21,26]. Contemporary clinical reports show that SQW is safe and effective in the treatment of senile dementia and can significantly improve the cognitive function and behavior of patients [27]. Experimental studies have shown that SQW can improve the cognitive impairment of mice induced by D-galactose [28], reduce Aβ deposition and neuronal damage in the hippocampus of AD model rats [29], and inhibit the apoptosis of neurotrophin-3 positive cells [30], thus playing a role in the prevention and treatment of AD. Paeonol in cortex paeonifolia elevates cortical cytochrome oxidase and vascular actin levels and improved behavior in a rat model of Alzheimer’s disease [31]. Cornus iridoid glycosides (CIG) can reduce the hyperphosphorylation of Tau protein and the deposition of pathological Tau protein in the amygdala region [32]. Our previous studies have also shown that SQW significantly increased glucocorticoid content and affected the expression of AKT/p-AKT protein in astrocytes. However, the effects of SQW on the PI3K-AKT pathway in AD have not been clarified, and would involve the further application of SQW.

Streptozotocin (STZ), which is used to destroy the insulin signal transduction pathway, can simulate a sporadic AD model by injection into the lateral ventricle (ICV) [33]. Therefore, we chose ICV-STZ to study the improvement effect of SQW on cognitive function in rats. In this study, we also combined transcriptomic analysis and biomedical experiments to determine the potential mechanism of SQW in AD rats induced by STZ.

## 2. Materials and Methods

### 2.1. Animals

A total of 50 adult male SD rats, aged 6 weeks old, (weighing approximately 220 ± 10 g) were purchased from Shanghai Super-B&K Laboratory Animal Corp., Ltd. (SCXK (Hu) 2018–0006, Shanghai, China), and all rats were uniformly housed in the Zhejiang Chinese Medical University Laboratory Animal Research Center under standard laboratory condition with an appropriate temperature of 23 ± 2 °C, 50–60% relative humidity, and a 12 h light-dark cycle. This animal experiment program was approved by the Ethics Committee of Zhejiang Chinese Medical University (Approval No.: IACUC-20210607-05).

### 2.2. Drugs and Reagents

SQW was purchased from Henan Wanxi Pharmaceutical Co., Ltd. (production batch number: 200403), and consisted of eight medicinal materials: Radix Rehmanniae (160 g), Chinese Yam (80 g), Cornus officinalis (80 g), Alisma Orientale (60 g), Poria Cocos (60 g), Cortex Moutan (60 g), Cassia Twig (20 g), and Aconite (20 g). The clinical equivalent dose of rats was calculated according to the conversion coefficient of human body surface area (60 kg for human, 200 g for rat). Weights of 75 g, 150 g, and 300 g of SQW were dissolved in distilled water and diluted to 500 mL to obtain SQW suspensions with doses of 1.5 g/kg, 3.0 g/kg, and 6.0 g/kg, respectively. The suspensions were then stored at 4 °C for subsequent experiments. STZ was purchased from Shanghai yuanye Bio-Technology Co., Ltd. (production batch number: 130,904). STZ was dissolved in sterile physiological saline at a concentration of 3 mg/kg.

### 2.3. Streptozotocin (STZ)-Induced Rat Model of AD and Treatment

**Figure 1:**
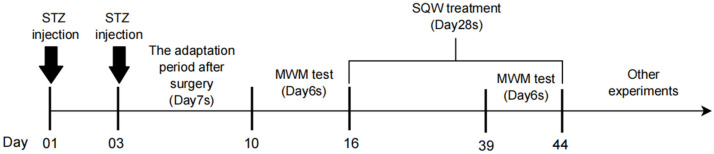
The schedule of animal experiments.

Rats were randomly divided into control group, AD induced group (ICV-STZ), treatment groups (AD disease plus SQW treatment at a dose of 1.5, 3.0, and 6.0 g/kg), and administration groups (1.5, 3.0, and 6.0 g/kg), with 10 rats in each group. They were adapted to feeding for 1 week and fasting for 12 h and water for 4 h before surgery. Rats were anesthetized with sodium pentobarbital (0.15 mL/100g, Sigma, Darmstadt, Germany) and then placed on the brain stereotaxic apparatus (Stoelting, Wood Dale, IL, USA). On the first and third days, the model group and the SQW (Henan Wanxi Pharmaceutical Co., Ltd., Henan, China) group of the rats were injected with 5 μL STZ (3 mg/kg, Shanghai yuanye Bio-Technology Co., Ltd., Shanghai, China) in the lateral ventricle with a micro-syringe at a rate of 0.5 μL/min with the following coordinates: 0.8 mm posterior to the bregma, 1.5 mm to the right side of the sagittal suture, and 3.6 mm below to the brain surface [33,34]. The needle remained for 5 min after injection, and was then slowly withdrawn. Rats of the control group were injected with the same volume of sterile normal saline. After 7 days of surgery, the learning and memory ability of the rats was estimated with a Morris water maze (MWM) test. Subsequently, the treatment groups were given 1.5 g/kg, 3.0 g/kg, and 6.0 g/kg SQW by gavage once a day for 28 days. The control and model groups were given the same amount of sterile 0.9% saline. After the treatment of SQW, three rats were randomly selected from each group and sacrificed, and the others were used for the MWM test. Subsequently, the brain samples were collected and stored at −80 °C for the following experiments (Figure 1).

### 2.4. Morris Water Maze Test

The MWM test was conducted in a circular pool with a diameter of 180 cm (Harvard Apparatus, Smart-Mass00916s, Cornellà (Barcelona, Spain) to evaluate the effect of SQW on learning and memory impairment in AD rats. Water was injected into the pool, with the water surface about 1cm higher than the platform, and titanium dioxide was added to dye the water white. The water temperature was controlled at 25 ± 2 °C. The pool was divided into four quadrants, and a transparent platform was settled in the fourth quadrant with a 1 cm depth below the water surface. The positioning navigation experiment was carried out twice a day for five days. In each training, a water entry point was randomly selected, and the time for each group of rats to find the platform was recorded. If the rats could not complete the training alone, they could search for the platform under our guidance, and then be allowed to stay on the platform for 15 s. On the sixth day, the platform was removed, and the rats were placed in the pool facing the wall of the tank and allowed to search the platform for 60 s. Finally, their swimming trajectory, the time proportion of the quadrant where the platform was located, and the swimming speed were recorded.

### 2.5. Hematoxylin and Eosin (H&E) and Nissl Staining

After SQW administration for 4 weeks, three rats were randomly selected from each group, intraperitoneally anesthetized with 3% sodium pentobarbital (0.15 mL/100 g), and perfused with 4% paraformaldehyde, and the brain tissue was taken out, fixed in 4% paraformaldehyde solution for 24 h, and then sequentially dehydrated, cleaned, dipped in wax, and embedded in sections. The paraffin sections were dewaxed to water, stained with hematoxylin, differentiated with hydrochloric acid and ethanol, washed with water, blued, counterstained with eosin solution, and incubated with toluidine staining solution for 30 min to stain neuronal Nissl bodies [35,36]. The morphological changes of neurons in the hippocampal CA3 area were observed under a Zeiss upright microscope (AXIO SCOPE.A1, CarlZeissJena, Oberkochen, Germany).

### 2.6. Immunohistochemistry Analysis

After dewaxing, hydration, and antigen retrieval in xylene, paraffin sections were supplemented with an antibody against NeuN (1:200, Huaan Biotechnology, Hangzhou, China), a marker of hippocampal neurons, developed with DAB, and then counterstained with hematoxylin. Subsequently, the loss of NeuN in the hippocampal CA3 region was observed on a Zeiss upright microscope, and the expression of NeuN in the brain slices of three rats was quantitatively analyzed using Image-Pro Plus 6.0 software.

### 2.7. RNA-Sequencing

The hippocampus tissue of three rats in the control group, the model group, and the SQW group (6.0 g/kg) was randomly selected as samples, and RNA sequencing was performed on them by Lianchuan Biotechnology Co., Ltd. (Hangzhou, China). Differentially expressed mRNAs (De mRNAs) were screened with |Fold Change| ≥ 2 or ≤ 0.5 and *p*-value < 0.05 as screening criteria. Gene Ontology (GO) analysis and the Kyoto Encyclopedia of Genes and Genomes (KEGG) pathway analysis were performed using the Database for Annotation, Visualization, and Integrated Discovery (DAVID, https://david.ncifcrf.gov/summary.jsp, accessed on 18 July 2021). Furthermore, the GO terms and KEGG pathways with an adjusted *p*-value < 0.05 and enriched genes ≥ 1 were defined as significant GO terms and KEGG pathways in the DAVID analysis according to a previous study [37,38]. Differential genes related to insulin resistance PI3K/AKT were screened out, and further analysis was performed by Cytoscape software (a PPI network visualization analysis platform) in order to evaluate their functional associations. Proteins with an interaction score > 0.4 were considered statistically significant.

### 2.8. qRT-PCR

The expression levels of five differential genes were detected by qRT-PCR assay. Firstly, TRIzol Reagent™ (Sigma, Darmstadt, Germany) was used to extract total RNA in the hippocampal homogenate, and a 30μL reverse transcription system was prepared according to TaKaRa reverse transcription kit (TaKaRa, Kyoto, Japan) to reverse transcribe RNA into cDNA. The instructions of a TaKaRa fluorescence quantitative kit (TaKaRa, Kyoto, Japan) were strictly followed. Quantitative Real-time PCR was performed, and β-Actin (Shanghai Sangon Bioengineering Co., Ltd.) was used as an internal control. The primer sequences were designed and synthesized by Shanghai Sangon Bioengineering Co., Ltd. (Shanghai, China). The primer sequences used in this study are shown in Table 1.

### 2.9. Western Blot Analysis

The rat hippocampus tissue was added to 150 mL of lysis buffer (RIPA:PMSF:protease inhibitor:protein phosphatase inhibitor = 100:1:1:1), and the tissue was homogenized with a homogenizer (ULTRA-TURRAX, IKA, Schwarzwald, Germany). The hippocampus tissue was then lysed for 30 min and centrifuged at 13,000 rpm for 15 min. The protein content in the supernatant was determined according to the instructions of the BCA kit (Beyotime, Shanghai, China). Protein samples were separated by 10% SDS-polyacrylamide electrophoresis gels, transferred onto polyvinylidenedifluoride (PVDF) membrane, and blocked with 5% BSA for 1 h. The membranes were incubated with primary antibodies: P-tau, tau, Caspase-3, PI3K, p-AKT, Total AKT (1:1000, Cell Signaling, Danvers, MA, USA), Bcl-2, GSK-3β (1:1000, Abcam, Cambridge, UK), NeuN, PDK1, Bax, GAPDH (1:1000, Huaan Biotechnology, Hangzhou, China), and α-Tublin (1:1000, BOSTER, Pleasanton, CA, USA) overnight at 4 °C, followed by incubation with goat against rabbit/mouse HRP-conjugated secondary antibodies (1:5000, immunoway, Plano, TX, USA) for 1 h at 37 °C. An ECL luminescence developing solution (A solution: B solution = 1:1) was then prepared, taking care that that light exposure was avoided. After adding 100 μL of developing solution to each band, it was placed on a chemiluminescence detector (Quick Chemi 5200, Monad, Suzhou, China) to scan the band.

### 2.10. Statistical Analysis

Results are expressed as means ± standard deviation (SD). SPSS 22.0 statistical software was used to process the experimental data. Analyses were performed using One-way ANOVA followed by Tukey’s tests. *p* < 0.05 was considered statistically significant. Figures were obtained using GraphPad Prism 8 (GraphPad Software, San Diego, CA, USA).

## 3. Results

### 3.1. SQW Improves Learning and Memory Impairment in STZ-Induced AD Rats

**Figure 2:**
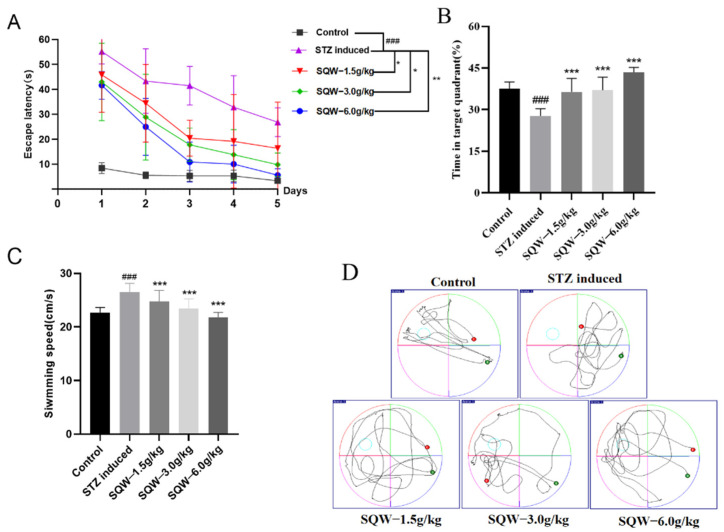
SQW improves STZ-induced learning and memory impairment in rats (*n* = 7). (**A**) The results of escape latency in five groups. (**B**) The proportion of time in the platform quadrant. (**C**) Average swimming speed. All of the results are expressed as means ± SD, *n* = 7 per group. Compared with the control group, ^###^ *p* < 0.001; compared with the STZ induced group, * *p* < 0.05, ** *p* < 0.01, *** *p* < 0.001. (**D**) Swimming trajectory graph.

To investigate the effect of SQW on learning and memory in STZ-induced rats, the Morris water maze test was performed after SQW administration for 28 days. With the increase of experimental training days, the escape latency of experimental rats in each group gradually shortened. However, compared with the control group, the escape latency of the rats in the model group increased significantly, while the escape latency decreased significantly after SQW administration (Figure 2A). In the space exploration experiment, the proportion of time in the quadrant where the platform was located was significantly reduced in the model group, and the swimming speed was also relatively slow; compared with the rats in the model group, the proportion of time in the quadrant where the platform was located in the SQW administration group was significantly reduced, and swimming speed was also faster (Figure 2B,C). The above-described situation can be seen in the swimming trajectory graph (Figure 2D), which indicated that SQW could ameliorate spatial memory impairment in STZ-induced rats.

### 3.2. SQW Attenuates STZ-Induced Pathological Damage in the Hippocampus

**Figure 3:**
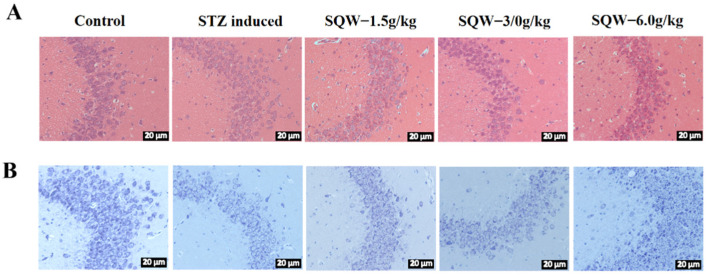
SQW alleviates the pathological damage of the hippocampus induced by STZ in rats. (**A**) The effect of SQW on the neuronal morphology of the hippocampal CA3 region induced by STZ in rats (Scale bar = 20 μm). (**B**) The effect of SQW on Nissl bodies of the hippocampal CA3 region induced by STZ in rats (Scale bar = 20 μm).

The results showed that SQW could alleviate the pathological damage of the hippocampus induced by STZ-induced rats. As shown in Figure 3A, the HE staining showed that the neurons in the hippocampal CA3 area of the rats in the control group were neatly and densely arranged, with regular morphology and darker coloration. In the control group, the neurons were arranged neatly and tightly, with no obvious gaps between the neurons, the neurons were round, the nucleoli were clear, and there was no shrinkage. In the model group, the neurons were abnormal and loosely arranged, the neurons volume was significantly reduced and shrunken, and the structure was blurred. After SQW treatment, it was obviously improved.

The results of Nissl staining are shown in Figure 3B. Compared with the control group, ICV-STZ-induced neurons in the CA3 area had a scattered arrangement and large gaps, blurred Nissl bodies, lighter staining, and significantly lower Nissl body cells. The morphology of neurons in the CA3 region of the hippocampus of the rats in the SQW administration group was significantly improved, and the number of Nissl bodies was significantly increased.

### 3.3. SQW Improves NeuN Neuron Loss and Tau Hyperphosphorylation in the Hippocampal CA3 Region

**Figure 4:**
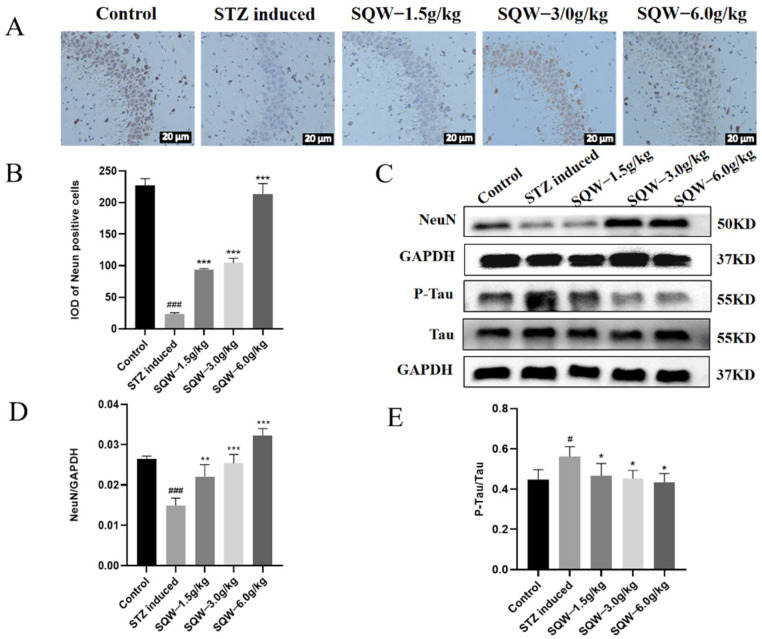
SQW improves NeuN neuron loss and Tau hyperphosphorylation in the hippocampal CA3 region in STZ-induced rats. (**A**) Immunohistochemical staining results of NeuN protein in hippocampal CA3 region. (**B**) Statistical results of IOD of hippocampal NeuN protein integral optical density in hippocampal CA3 region. (**C**) Protein bands for NeuN, Tau, and p-Tau. (**D**) The effect of SQW on NeuN protein expression. (**E**) The Effect of SQW on the phosphorylation level of Tau protein. All of the results are expressed as means ± SD, *n* = 3 per group. Compared with the control group, ^#^ *p* < 0.05, ^###^ *p* < 0.001; compared with the STZ induced group, * *p* < 0.05, ** *p* < 0.01, *** *p* < 0.001.

The results of immunohistochemistry in Figure 4A,B showed that the NeuN positive cells in the model group were sparsely arranged, and the number of cells was less when compared with the control group. The results of Western blot quantitative analysis also showed that the expression level of NeuN protein in the model group was significantly decreased, and its expression level was reversed after SQW administration (Figure 4C,D). We also quantitatively analyzed the phosphorylation level of Tau protein by Western blot analysis. Compared with the model group, the ratio of p-Tau/Tau in the STZ group was significantly reduced (Figure 4E). The above results indicate that SQW may exert a neuroprotective effect by preventing the loss of NeuN neurons and the hyperphosphorylation of Tau in hippocampus tissue of STZ-induced rats.

### 3.4. SQW Improves the Level of Apoptosis in STZ-Induced AD Rats

**Figure 5:**
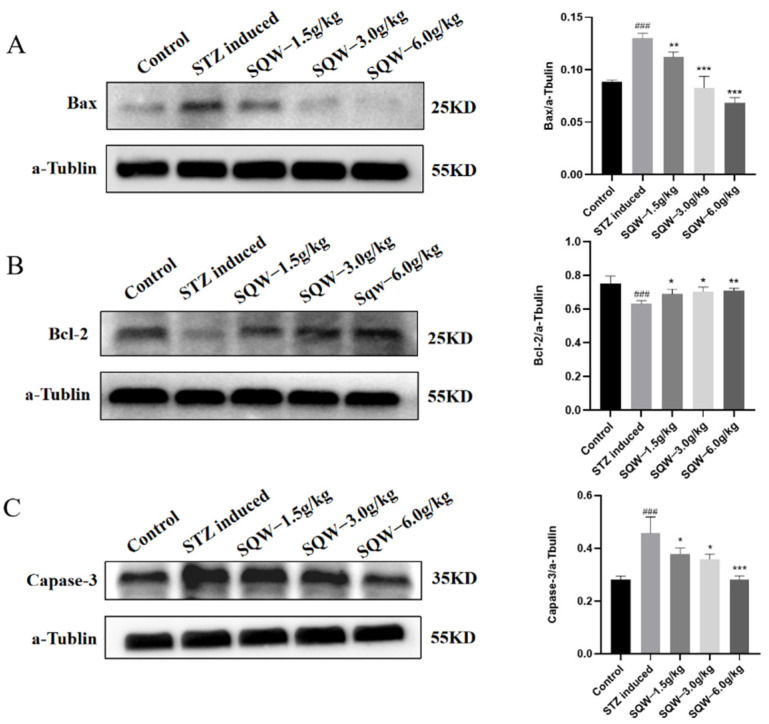
The effect of SQW on the protein expression levels of Bax, Bcl-2, and Caspase-3. (**A**) The effect of SQW on Bax. (**B**) The effect of SQW on Bcl-2. (**C**) The effect of SQW on Caspase-3. All of the results are expressed as means ± SD, *n* = 3 per group. Compared with the control group, ^###^ *p* < 0.001; compared with the STZ induced group, * *p* < 0.05, ** *p* < 0.01, *** *p* < 0.001.

To explore the effect of SQW on apoptosis, Western blot was used to detect the protein expression levels of Bax, Bcl-2, and Caspase-3. The results showed that after ICV-STZ, the expressions of Bax and Caspase-3 in the model group were significantly increased, and the expression level of Bcl-2 was significantly decreased compared with the control group. However, after administration of SQW for 28 days, the level of anti-apoptotic signaling molecules (Bcl-2) was decreased and the level of pro-apoptotic signaling molecules (Bax) and protease (Caspase-3) were increased (Figure 5). SQW may protect STZ-induced AD rats by reversing the level of apoptosis.

### 3.5. SQW Regulates the Differential Expression of Hippocampal mRNA in STZ-Induced Rats

**Figure 6:**
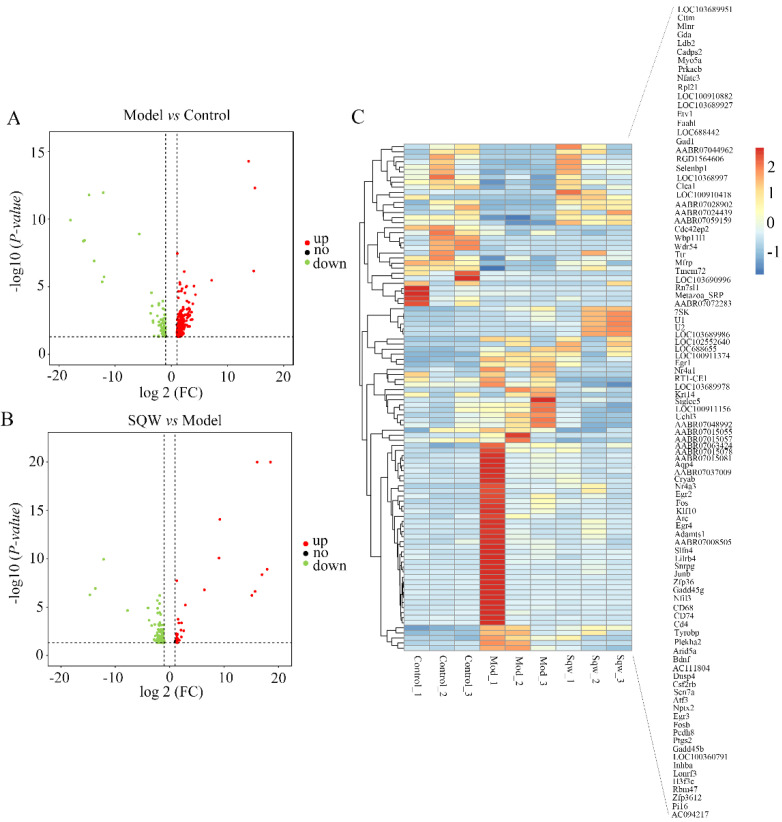
Identification of De mRNAs in AD rats with SQW treatment. (**A**) Volcano plot for De mRNAs between STZ induced group and control group. (**B**) Volcano plot for De mRNAs between SQW group and model group. The horizontal axis is logFold Change (FC), and the vertical axis is the *p*-value after the negative logarithm conversion. Up- and down-regulated genes are marked in red and blue, respectively. (**C**) Heat map of the top 100 differentially expressed genes. Red indicates high expression levels and green low expression levels.

**Figure 7:**
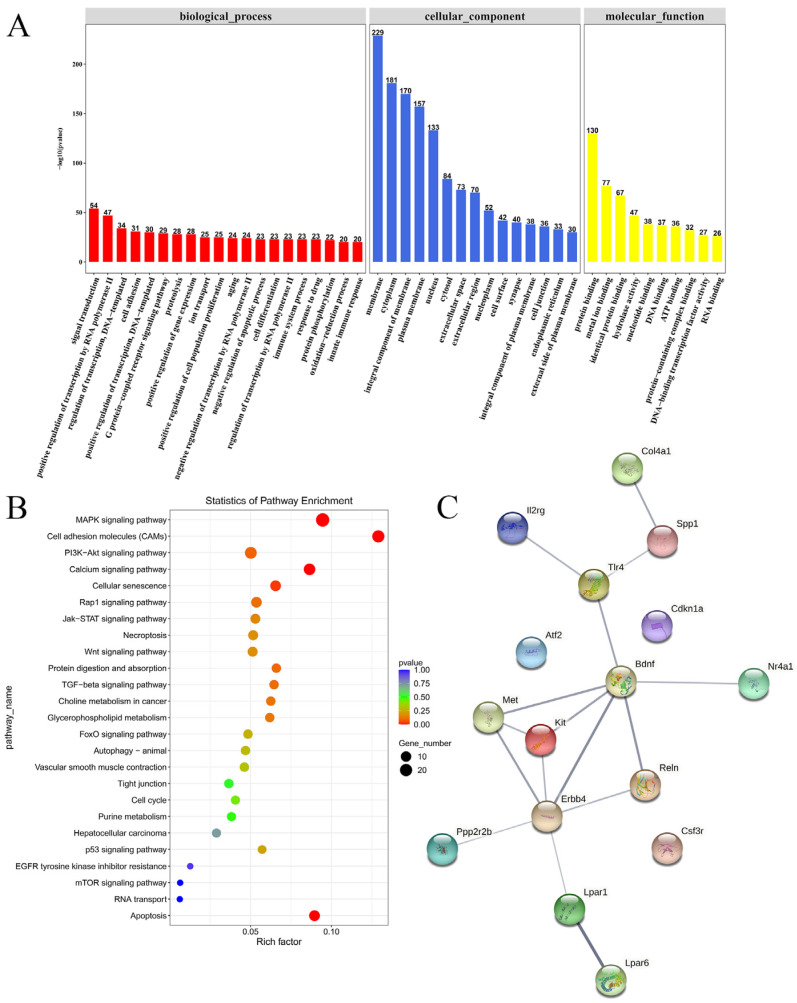
Functional enrichment analysis and PPI network. (**A**) GO terms of the biological processes, molecular function, and cell component enriched by DE mRNAs. (**B**) KEGG pathways enriched by DE mRNAs among the different groups (*p* < 0.05). The rich factor is defined as the ratio of the number of differentially expressed mRNAs and the number of genes in the corresponding KEGG pathways, and larger values represent a greater degree of pathway enrichment. (**C**) Protein–protein interaction (PPI) network of PI3K/AKT pathway-related targets.

RNA-sequencing data showed that there were 344 differentially expressed mRNAs (264 up-regulated and 80 down-regulated) after STZ injection, and a total of 194 gene expression changes after SQW treatment (155 up-regulated and 39 down-regulated). These differentially expressed mRNAs are shown in hierarchical volcano maps and heat maps in Figure 6. After further biological analysis, GO analysis showed that the De mRNAs were mainly distributed in the cell membrane, cytoplasm, and nucleus. This involves a wide range of molecular functions and biological processes. The main related biological processes include the phosphorylation of DNA templates, nucleotide binding, ATP binding, etc. We also used KEGG to further analyze the signaling pathways involved in differential genes, and the De mRNAs were mainly enriched in the MAPK signaling pathway, PI3K/AKT signaling pathway, TNF signaling pathway, and calcium ion signaling pathway. PI3K/AKT is a classic insulin resistance signaling pathway. A number of studies on the pathogenesis of AD have pointed out that insulin resistance is closely related to hyperphosphorylation of tau protein, β-amyloid plaque deposition, and neuronal apoptosis. It is considered to be one of the important pathogenesis of AD. Therefore, the differential genes related to the PI3K/AKT pathway were further screened: Atf2, Ppp2r2b, Nr4a1, Bdnf, and Lpar1; the protein–protein interaction (PPI) network of the PI3K/AKT pathway-related targets is shown in Figure 7C.

### 3.6. Transcriptomic qRT-PCR Validation Results

**Figure 8:**
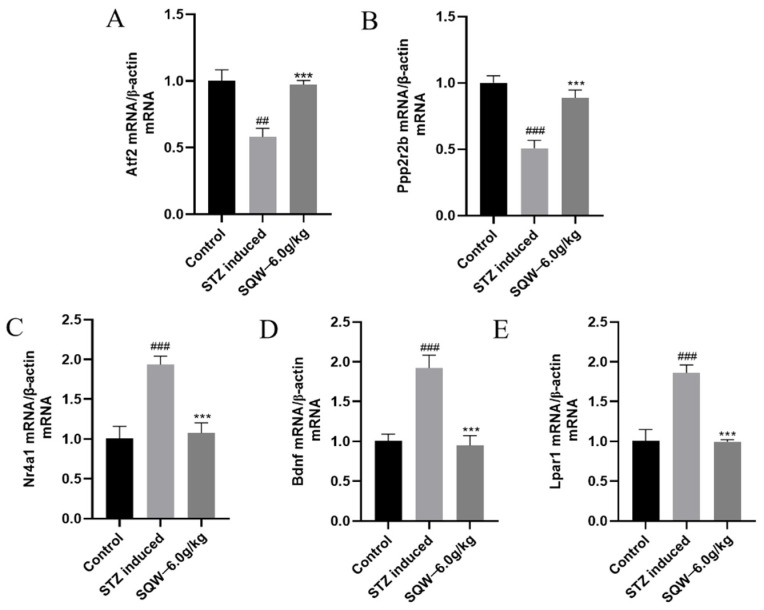
Insulin resistance-related genes in the hippocampus of AD rats treated with STZ. (**A**,**B**) Two genes, Atf2 and Ppp2r2b, were significantly increased, and the gene expression levels were reversed after SQW (6.0 g/kg) treatment. (**C**–**E**) The genes of Nr4a1, Bdnf, and Lpar1 were significantly decreased, and the gene expression levels were reversed after SQW (6.0 g/kg) administration. All of the results are expressed as means ± SD, *n* = 3 per group. Compared with the control group, ^##^ *p* < 0.01, ^###^ *p* < 0.001; compared with the STZ induced group, *** *p* < 0.001.

The De mRNAs (Atf2, Ppp2r2b, Nr4a1, Bdnf, Lpar1) were verified by qRT-PCR assay. As shown in Figure 8, compared with the control group, the Atf2 and Ppp2r2b mRNA levels in the model group were significantly decreased (Figure 8A,B) and the mRNA levels of Nr4a1, Lpar1, and Bdnf were significantly increased (Figure 8C–8E). After SQW treatment (6.0 g/kg) for 28 days, the mRNA levels of the five differential genes were reversed. For the verification results, it can be seen that the expression trend of the De mRNAs was the same as that of the results of RNA sequencing.

### 3.7. SQW Regulates the Expression Levels of PI3K/AKT Signaling Pathway-Related Proteins in STZ-Induced Rats

**Figure 9:**
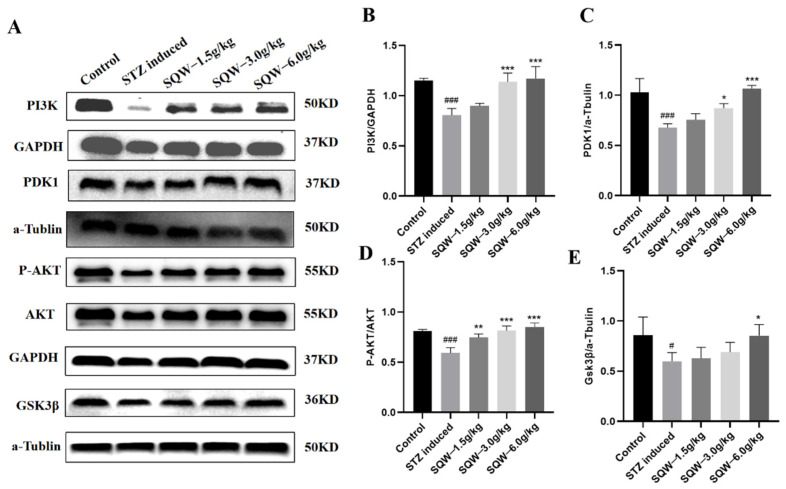
The effects of SQW on the protein expression level of PI3K/AKT signaling pathway. (**A**) Band diagram of PI3K pathway-related proteins. (**B**) The effect of SQW on the expression level of PI3K protein. (**C**) The effect of SQW on the expression level of PDK1 protein. (**D**) The effect of SQW on the phosphorylation level of AKT protein. (**E**) The effect of SQW on the expression level of GSK3β protein. All of the results are expressed as means ± SD, *n* = 3 per group. Compared with the control group, ^#^ *p* < 0.05, ^###^ *p* < 0.001; compared with the STZ induced group, * *p* < 0.05, ** *p* < 0.01, *** *p* < 0.0001.

The PI3K/AKT pathway is a classic insulin resistance signaling pathway, which is thought to be altered during the development of AD [39]. In order to explore the mechanism by which SQW improves STZ-induced cognitive impairment, we detected PI3K, PDK1, p-AKT, AKT, and GSK3β expression. Compared with the control group, the expression levels of PI3K, PDK1, p-AKT, and GSK3β in the STZ group were significantly decreased, while the expressions of PI3K, PDK1, p-AKT, and GSK3β were increased after treatment with different doses of SQW. The results showed that the PI3K/AKT pathway played an important role in the STZ-induced AD model, and SQW maintained the function of the insulin signaling pathway in the brain (Figure 9).

## 4. Discussion

AD is a neurodegenerative disease positively correlated with age and related to memory, cognitive impairment, and behavioral changes. At present, its pathogenesis is not clear [1]. Epidemiological studies in recent years show that AD is closely related to metabolic diseases, and T2DM can significantly increase the risk of dementia [40]. Moreover, AD and T2DM have common pathological features, such as Aβ deposition, tau hyperphosphorylation, inflammatory reaction, insulin deficiency and resistance, and signal system disorder [41,42], among which one of the most important features is insulin resistance and its signal transduction damage [43,44]. Previous studies have found that STZ can trigger type 1 and type 2 diabetes by destroying pancreatic β cells and inducing insulin resistance [45,46]. In contrast, the pathophysiology of the ICV-STZ animal model is similar to that of AD patients [47]. Furthermore, ICV-STZ has now been found to induce cognitive impairment and neuron damage [48], oxidative stress [49] and glucose/energy metabolism damage in the brain [50], insulin resistance in the brain [51,52], and finally lead to Tau hyperphosphorylation and Aβ deposition [53,54].

In this study, as previously reported, ICV-STZ-induced AD rats showed learning and memory impairments in the MWM test. They had a longer escape latency in the positioning navigation experiment and had little access to the dismantled platform area in the space exploration test. However, the SQW treatment group showed significant improvement in the Morris water maze test. These rats had shorter escape latency, spent the most time in the target quadrant, and swam faster, and the improvement effect of the 6.0 g/kg SQW group was the most significant. This indicated that SQW could improve the learning and memory ability of AD rats, and the dosage of 6.0 g/kg was more efficient than other doses. Moreover, the expression of NeuN and the phosphorylation of Tau protein were significantly reversed in the treatment group.

Apoptosis, as an essential factor in the pathological process of AD, has also received significant attention in recent years [55,56]. Researchers have observed the expression of apoptosis-related proteins and nuclear apoptosis bodies in neurons in brain autopsies of AD patients, and apoptosis may be part of the mechanisms of AD neuron loss [57]. Bcl-2 is an anti-apoptotic gene that plays an integral role in regulating the endogenous mitochondrial apoptosis pathway [58]. Bax is a pro-apoptosis protein that can change the permeability of the mitochondrial outer membrane and eventually induce the endogenous apoptosis of cells [59]. Related studies have found that the content of Bcl-2 decreased significantly, and the content of Bax is significantly increased in nerve cells of AD animal models [60,61]. Caspase-3, as the downstream of the apoptosis cascade, is considered the most important executor of inducing apoptosis, and its expression level can directly reflect the degree of apoptosis [62]. Studies have shown that caspase-3 participates in the cleavage of Tau protein, and the cleaved Tau protein becomes the effector of apoptosis [63]. In this study, we observed a significant increase in Bax and caspase-3 and a significant decrease in anti-apoptosis factor (Bcl-2) in rats induced by ICV-STZ compared with the control group. After SQW treatment, we observed a decrease in Bax/Bcl-2 ratio and a reversal of down-regulation of caspase-3 expression. Among the three doses of SQW, high-dose SQW had the best anti-apoptosis effect, consistent with the test results on learning and memory ability.

Insulin is an important hormone that regulates the metabolism of the body. The level of insulin in the brain can directly affect its substance and energy metabolism, and then affect cognitive function. Insulin plays a normal metabolic role in the brain, which needs to combine with the α subunit of InsR, cause autophosphorylation of the β subunit, and activate IRS1, and then activate the PI3K/AKT insulin signaling pathway [12,13,64]. Activated Akt kinase subsequently phosphorylates the Akt substrate 160 kDa (AS160), recruiting insulin-dependent glucose transporter 4 (GLUT4) to the plasma membrane, allowing the efficient entry of glucose into cells [65]. Studies have found that IR exists in the brain of AD patients. Long-term IR in the brain will seriously damage the PI3K/AKT signal level pathway, resulting in the disorder of the expression of GSK3β and mTOR, which will further destroy the steady-state of brain energy metabolism, induce the deposition of Aβ and hyperphosphorylation of Tau protein, and thus cause cognitive impairment [66,67]. Therefore, in this study, we analyzed a set of genome-wide RNA sequencing data from the GEO database, and finally screened five genes, Atf2, Ppp2r2b, Nr4a1, Bdnf, and Lpar1, which are different from the PI3K/AKT pathway. The differential expression of these five genes in STZ-induced AD rats was then confirmed by qRT-PCR, and the gene expression levels were reversed after treatment with SQW (6.0 g/kg). The quantitative analysis of PI3K/AKT signal pathway-related proteins by Western blot then showed that, compared with the control group, the expression of PI3K, PDK1, p-AKT, and GSK3β proteins in the STZ-induced group was significantly down-regulated. The expression level of these proteins was up-regulated significantly after the intervention of SQW, and the improvement effect of the SQW (6.0 g/kg) group was the most significant.

Most insulin metabolic activities are activated through the PI3K pathway, and when any link in this signaling pathway is weakened, it may inhibit the physiological effect of insulin and lead to IR. For example, GLUT4 is a key protein related to glucose transport downstream of the IRS-1/PI3K pathway. Phosphorylation of AKT can accelerate the transfer of GLUT4 from the cell to the cell membrane, where it fuses with the cell membrane and increases glucose uptake [68,69]. In contrast, insulin-regulated GLUT4 trafficking is mediated by the formation of specific SNARE complexes [70,71]. Recent studies have found that disturbances in SNARE complex function can alter steroid hormone production and central nervous system behavior, as well as peripheral metabolism, providing potential insights into the relationship between metabolic and neuropsychiatric disorders in humans. It also suggests that this is a direction we may explore in depth in the future [72].

To sum up, SQW may reverse the damaged PI3K/AKT signaling pathway, improve the learning and memory impairment induced by STZ in AD rats, and inhibit neuronal apoptosis, thus playing a preventive and therapeutic role in AD rats.

## 5. Conclusions

In conclusion, our results suggested that SQW could contribute to neuroprotection against learning and memory impairment in rats induced by STZ through activation of the PI3K/AKT signaling pathway. This study is expected to provide an experimental basis for the scientific connotation of SQW in the prevention and treatment of AD.

## Figures and Tables

**Table 1:** Sequences of primer for qRT-PCR.

Primer	Sequence 5′-3′	Primer Length
Bdnf-F	AGGCTTGACATCATTGGCTGACAC	24 bp
Bdnf-R	GGCACTTGACTACTGAGCATCACC	24 bp
Ppp2r2b-F	CCGCTGATGACCTGAGGATTAACC	24 bp
Ppp2r2b-R	GTGGAACTCGGCTGCTGTGATC	22 bp
Lpar1-F	AATCTATGTCAACCGCCGCTTCC	23 bp
Lpar1-R	GCCATGTGCTAACAGTCAGTCTCC	24 bp
Nr4a1-F	GCACCTTCATGGACGGCTACAC	22 bp
Nr4a1-R	CTGAGGACGAGGATGTGGAGGAG	23 bp
Atf2-F	GGTCATGGTAGCGGATTGGTTAGG	24 bp
Atf2-R	GTAGTGGATGTGGCTGGCTGTTG	23 bp
Kit-F	GCGTTCTGCTCCTACTGCTTCG	22 bp
Kit-R	TGGATGGATGGTGGAGACGGTTC	23 bp
β-Actin-F	TCAGGTCATCACTATCGGCAAT	22 bp
β-Actin-R	AAAGAAAGGGTGTAAAACGCA	22 bp

## Data Availability

The datasets used and/or analyzed during the current study are available from the corresponding author on reasonable request.

## Abbreviations

AD	Alzheimer disease
SQW	Shen Qi wan
Aβ	Amyloid-beta
Tau	microtubule-associated protein
T2DM	type 2 diabetes mellitus
IR	insulin resistance
PI3K/AKT	phosphatidylinositol 3-kinase/protein kinase B
PDK	PI3K-dependent kinase
APP	amyloid precursor protein
TCM	Traditional Chinese Medicine
CIG	Cornus iridoid glycosides
STZ	Streptozotocin
SD rat	Sprague-Dawley rat
ICV	intracerebroventricular
MWM	Morris water maze test
HE	Hematoxylin-eosin staining
mRNA	Messenger ribonucleic acid
qRT-PCR	quantitative Real-time PCR
WB	Western Blot
De mRNAs	Differentially expressed mRNAs
GO	Gene Ontology
KEGG	Kyoto Encyclopedia of Genes and Genomes
